# What can we learn from simulation-based training to improve skills for end-of-life care? Insights from a national project in Israel

**DOI:** 10.1186/s13584-017-0169-9

**Published:** 2017-11-06

**Authors:** Mayer Brezis, Yael Lahat, Meir Frankel, Alan Rubinov, Davina Bohm, Matan J Cohen, Meni Koslowsky, Orit Shalomson, Charles L Sprung, Henia Perry-Mezare, Rina Yahalom, Amitai Ziv

**Affiliations:** 1 Center for Quality and Safety, Hadassah Medical Center & Braun School of Public Health, Hebrew University, Jerusalem, Israel; 2 Clalit Health Services, Tel Aviv, Israel; 3 Hadassah Medical Center & Hebrew University, Jerusalem, Israel; 4 Department of Psychology, Bar-Ilan University, Ramat Gan, Israel; 5 Israel Center for Medical Simulation (MSR), Chaim Sheba Medical Center, Tel Hashomer, Israel & Sackler School of Medicine, Tel-Aviv University, Tel-Aviv, Israel

**Keywords:** End-of-life, Simulation-based training, Simulated patients, Standardized patients, Actors, RIAS

## Abstract

**Background:**

Simulation-based training improves residents’ skills for end-of-life (EOL) care. In the field, staff providers play a significant role in handling those situations and in shaping practice by role modeling. We initiated an educational intervention to train healthcare providers for improved communication skills at EOL using simulation of sensitive encounters with patients and families.

**Methods:**

Hospital physicians and nurses (*n* = 1324) attended simulation-based workshops (*n* = 100) in a national project to improve EOL care. We analyzed perceptions emerging from group discussions following simulations, from questionnaires before and after each workshop, and from video-recorded simulations using a validated coding system. We used the simulation setting as a novel tool for action research. We used a participatory inquiry paradigm, with repetitive cycles of exploring barriers and challenges with participants in an iterative pattern of observation, discussion and reflection – including a description of our own responses and evolution of thought as well as system effects.

**Results:**

The themes transpiring included lack of training, knowledge and time, technology overuse, uncertainty in decision-making, poor skills for communication and teamwork. Specific scenarios demonstrated lack of experience at eliciting preferences for EOL care and at handling conflicts or dilemmas. Content analysis of simulations showed predominance of cognitive utterances - by an order of magnitude more prevalent than emotional expressions. Providers talked more than actors did and episodes of silence were rare. Workshop participants acknowledged needs to improve listening skills, attention to affect and teamwork. They felt that the simulation-based workshop is likely to ameliorate future handling of EOL situations.

We observed unanticipated consequences from our project manifested as a field study of preparedness to EOL in nursing homes, followed by a national survey on quality of care, leading to expansion of palliative care services and demand for EOL care education in various frameworks and professional areas.

**Conclusions:**

Reflective simulation exercises show barriers and paths to improvement among staff providers. When facing EOL situations, physicians and nurses use cognitive language far more often than emotions related expressions, active listening, or presence in silence. Training a critical mass of staff providers may be valuable to induce a cultural shift in EOL care.

**Electronic supplementary material:**

The online version of this article (doi:10.1186/s13584-017-0169-9) contains supplementary material, which is available to authorized users.

## Background

Healthcare facing end-of-life (EOL) raises challenges of overuse of invasive technology, underuse of palliation, suffering of patients and families, serious dilemmas, conflicts and heavy costs [1]. Many people are treated at the EOL with intensive life-support modalities, without adequate discussion of the limited benefits of those options and of their potential harms, with reduced quality of life at the twilight of life [2, 3]. While discussing poor quality of care at the EOL [1], the literature misses a clear definition of the problem with valid and reliable criteria for measuring its magnitude. EOL itself eludes a practical definition [4]: while prognosis appears ominous, death is certain only in retrospect – when it is too late to change the care approach.

EOL care suffers from poor funding of palliative care, misaligned incentives, lack of integrated healthcare systems, and social isolation of elders – among the challenges that call for changes in policy, delivery and payment systems [1]. One important reason for the poor quality of care at EOL is deficient healthcare professional competencies in domains such as interpersonal skills, palliative care knowledge, teamwork, cultural proficiency, and ethics [1]. This relates to broader challenges: defining and assessing professionalism [5], applying these principles in residency training [6], and maintaining physician’s competence through lifelong learning [7].

To ensure competencies of healthcare workforce, simulation-based training is playing an increasingly significant role [8–10], including at EOL care, where studies have shown its effectiveness to improve communication skills [11–18]. Those studies have been conducted mostly with residents. Change in the quality of EOL care is lagging [19] in part perhaps because hospital senior staff, who attest having themselves communication difficulties [20, 21], may be those providing a more significant role than house officers in dealing with EOL and shape the team’s practice by leadership and role modeling.

The current paper describes the design and running of simulation-based workshops to improve EOL care, as well as insights from this experience.

## Methods

Five years ago, we initiated a national project to improve EOL skills – developing a workshop and inviting teams from all major hospitals in our country. We prepared the workshop for staff physicians, departments’ chiefs, head nurses and residents with help from physicians, psychologists, nurses, social workers and ethicists, to take place at the Israel Centre for Medical Simulation (MSR). MSR is an international leader in the innovative and evolving field of medical simulation and patient safety, providing multi-disciplinary training to health care professionals in a wide variety of vital domains, in over 60 courses, through facilitated hands-on practice in simulated medical environments [22]. The workshop used simulation with actors on EOL scenarios followed by facilitated video-based group discussion, and provided an opportunity to explore barriers and challenges for hospital staff in providing optimal care at the EOL.

The Israel Center for Medical Simulation [22] provided experience and logistics to develop and run a simulation-based workshop. A steering committee^1^ built six scenarios based on the following challenges: to elicit preferences for EOL care from a patient or from a relative; to handle conflicts between siblings or within the team; to handle requests to do “everything”; to explain whether to put in a feeding tube. The committee chose the number six as a compromise between the time constraints and the wish to cover common challenges related to EOL care, and the topics for the scenarios after deliberations derived from field experience and literature [23–25]. Several members of the steering committee (CS, AR and MB) have been interested for over a decade in the field of EOL. They have initiated formal and unformal ethics discussions on EOL cases and issues at staff meetings of their departments or institutional seminars and conferences. They have followed and/or contributed to the literature, and/or been involved in the preparation of the Dying Patient Act, and/or performed institutional quality projects with medical students at Hadassah-Hebrew University Medical Center on topics related to EOL care. These activities and experience were a natural preparation to the present project.

We trained professional actors to play the scenarios with options of responses to trainee’s behavior and to provide personal feedback as previously described [22].

All participants in the workshop came voluntarily and included physicians and nurses from internal medicine and geriatrics wards from all major hospitals in the country. To those who registered, we sent in advance copies of published papers [23–25], which describe tools we recommended to apply for handling EOL situations.

More information about the procedures for the workshops and the scenarios is available in Additional file 1: Appendix A (including additional adapted scripts with the extension of the program to other clinical settings as described below).

At the start, we believed we were going to train for communication skills [24] using previously described tools [11], and cope with the challenge of assessing the efficacy of an educational intervention for improved professional competence [5]. As we progressed over the years with the project, we became more and more aware than we were the ones who were learning. What is professional competence when facing EOL? What are the skills when mere human presence is an answer [26]? The concept of skills misrepresents communication, which is inherently creative [27]: skills and sincerity are inimical concepts [28]; can authenticity be inculcated [29]? The simulation-based workshops turned into a dynamic laboratory to explore barriers and challenges when healthcare teams face human finitude. The discussions brought up ideas related to the attachment theory [27], unlearning [30], paradoxical thinking [31] and tolerance of uncertainty [32]. The entire process raised more questions than answers – providing an unusual opportunity for reflection, deep learning and development. We found the simulation platform with deliberate practice [33]^2^ a useful reflective exercise for the complex learning associated with EOL care, as suggested by others [34, 35].

Using qualitative methodology, we analyzed themes and perceptions emerging from discussions and feedback taking place following simulations as done in focus groups or deliberative dialogue [36] – reframing simulation for engagement of stakeholders in improving EOL care [34, 35]. Action research integrates problem solving and theoretical inquiry while addressing an issue with those experiencing it, through collaborative learning and reflection in an ongoing cycle of co-generative knowledge [37]. In a sense, we have used the simulation setting as a novel tool for action research.

We complemented or triangulated those insights with information from questionnaires filled out by participants at the start and after each workshop (on skills and behavior; satisfaction from workshop, perceived challenges, changes in attitudes and open comments). The process of theoretical saturation in our study has been defined [36] as an iterative process of data collection and analysis, moving back and forth between emerging (from each new workshop) and existing data (from previous workshops) until we felt confident that no new information was likely to be revealed through additional data collection (and workshops).

We collected over 400 h of video-recorded workshop activities but it was beyond our resources capacity to have them transcribed and coded. The method we used has been described as “connecting strategy” for analyzing data generated through the deliberative dialogue that was going on in our workshops: [36, 38] “Connecting, or contiguity, strategies involve analysis of contextualized relationships, such as those employed in narrative inquiry. (…) Categorizing strategies alone, however, are sometimes criticized for decontextualizing and fragmenting data [38]. Context is a critical consideration for action-oriented health research, when the ultimate goal is informing policy and influencing practice within deeply contextualized health systems.” This mode of analysis appears suited for the dynamic paradigm of inquiry described below since coding is decontextualizing whereas inquiry is context sensitive.

The qualitative research paradigm we applied is probably best described as participatory inquiry paradigm [39, 40]. The major belief behind this paradigm is that since knowing is experiential, good research is collaborative research, i.e., with people rather than on people [41]. Although we initiated our project as a training program for EOL care, our interest shifted to deep understanding of the behavior, attitudes and knowledge of healthcare providers facing EOL. For instance, because of prognostic uncertainty in many critically illnesses, we became aware of the value of paradoxical thinking [31]. A key feature of participatory inquiry is the use inquiry cycles, in an iterative pattern between reflection and action. Over the years of our project, the workshops provided a remarkable platform for repeated cycles of reflection, sharing and discussing ideas, and attempted action in simulations. Interestingly, this paradigm has been implemented as cooperative action research in palliative care [42].

This methodology appears to comply largely with recommended criteria for trustworthiness of qualitative research: authenticity, comprehensiveness, credibility, integrity and responsiveness [43, 44, 36].

In addition, recorded simulations (*n* = 97) provided material for evaluation by the Roter Interaction Analysis System (RIAS) [45], using two trained coders (for predefined categories) with high inter-rater reliability (Cronbach alpha 0.9 tested for 10 random clips in the present study). The RIAS is a quantitative mean of analyzing data from dialogues in healthcare and simulations [46]. For statistical analysis, we applied ANOVA and Scheffé’s method for group comparisons, using SPSS version 19, IBM Corp, Armonk, NY. Sheba Medical Center IRB approved the study.

## Results

Since 2011, we have conducted 100 workshops,^3^ moderated by one of us (MB), for 1324 healthcare providers from 32 medical centers, including all major hospitals and 8 nursing homes in the country: Half of them physicians (25% residents), 47% nurses (nearly all RN) and 3% others (social workers, dieticians, clinical psychologists, physiotherapists or senior executives). Initiated as a limited, research training project, it became a national project in collaboration with Clalit Health Services (the largest health provider in Israel and owner of 14 hospitals, including some the largest in the country). After a successful workshop developed for teams of internal medicine and geriatrics, we extended the program with adapted scenarios to teams of nursing homes, dialysis and intensive care units, neurology, neurosurgery, family medicine and emergency rooms (see Additional file 1: Appendix A).

Data from the questionnaires showed that the participants often felt poorly equipped with skills needed for EOL care (see Additional file 2: Appendix B). The participants rated the simulations as representative reflection of field experiences and the play of the professional actors as so realistic that they often forgot the artificial setting. The participants also rated high the overall learning experience and most would strongly recommend it to other physicians and nurses. They believed that the simulation-based workshop was likely to ameliorate future handling of EOL situations (see details in Additional file 2: Appendix B).

The insights discussed below emerged over the years from the experience at the workshops that progressively reached theoretical saturation.

### Common themes

The following themes recurred throughout discussions and questionnaires at every workshop. *Lack of training*: A majority of participants admitted, “Never having received any education for EOL care”. *Lack of knowledge about the law*: Most participants said “they have poor knowledge of the Israeli Dying Patient Act” [47] as previously reported [48]. Only half of participants knew that this law requests staff to support patients’ families. We presented and discussed main principles of the law. *Lack of knowledge about palliative care:* Nearly two-third would not use opioids to relieve dyspnea for fear of respiratory depression (and few more would use nebulized opioids) despite the literature [49]. Many did not know that palliative care has been associated with improved survival [50]. We discussed those issues and others such as palliative sedation for refractory suffering, and encouraged participants to get help from local experts in palliative care. *Perception of overtreatment*: Most participants reported often providing futile care “they would not have wanted for themselves” or giving life-sustaining treatment in conditions they view as “associated with poor quality of life.” Other participants warned about “the subjectivity of quality of life judgments.” About 20% of them reported often performing “slow codes”. We discussed "the problem with futility" [51, 52]. *Dilemmas and uncertainty*: Many participants admitted being “often confused and perplexed about correct choices for patients”, while some would expect guidance from the regulation to determine “whether a patient is dying”. Others mentioned “miracles” (as described [53]) and an idea emerged that a dichotomized view of EOL (“dying or not dying”), attractive to Cartesian minds, is potentially risky (as in the Liverpool Care Pathway [54]) and could be replaced by more useful paradigm: paradoxical thinking, associated with better tolerance of uncertainty and creativity [31]. *Lack of time*: Many participants perceived lack of time as a major barrier in proper communication with patients and families: They complained that the 7-min simulation was too short, while others remarked, “in real-life we might not have even this luxury.” We discussed evidence showing that empathy is effectively expressed in less than 1 minute [55, 56] and that an affective channel of communication, (the primal way we learn to connect in infancy, towards which we regress in disease [57]) is much *faster* than cognitive messaging. Facial micro-expressions reflect emotions in less than one second [58], whereas to explain respiratory and renal failure from sepsis would probably require at least several minutes. *Lack of hope:* When facing EOL, participants felt bewildered by the recommendation “Communicating with hope” [24]. We discussed the multiplicity and subjectivity in the meaning of hope [59]: miraculous recovery, relief from suffering or closure and making peace at the end - a patient may view as “some of the best days in my life” [60]. While a clinician has difficulty seeing more than a grim prognosis, we proposed, as Surbone, reframing challenge from “truth telling to truth making” [61]: acknowledging uncertainty and committing to non-abandonment. *Poor teamwork culture*: At debriefing of simulations where a physician and a nurse handle a challenge together, participants reported, “Feeling uneasy and not used to multi-disciplinary meetings”, while some physicians were “reluctant to invite nurses to such meetings with patients or families.” Others stated they “routinely invite nurses or other team workers to join such meetings”: they believe that their presence sends a message of genuine concern, often assists in fine-tuning nuances in the conversation, and always help promoting the continuity of care. Specific scenarios demonstrated lack of experience at eliciting preferences for EOL care, and at handling conflicts or dilemmas (see Additional file 3: Appendix C). Altogether, participants acknowledged the need to improve listening skills, attention to affect and teamwork.

### Content analysis of video-clips using RIAS tool

The first 10 workshops took place in 2011 with 47 staff physicians, 33 residents and 40 nurses participating in six EOL scenarios (each ran twice), usually alone (sometimes in combination: physician with nurse, staff physician with resident). They provided 120 recorded 7-min simulation sessions and several hours of recorded debriefing sessions. Based on insights from the group discussions, we decided to evaluate the content analysis of video-clips using the RIAS tool under two classes of categories: *cognitive* and *affective*. We combined as *cognitive* utterances categories dealing with medical condition and therapeutic regimen (such as “data gathering” or “patient education and counseling”). We combined as *affective* utterances RIAS categories defined as “psychosocial issues”, “feelings”, and “building a relationship”. Analysis of video-clips showed predominance of cognitive utterances - by an order of magnitude more prevalent than affective expressions, as shown in Table 1. Males and physicians used more cognitive talk while females and nurses had a higher proportion of affective utterances. Providers talked far more often than actors did by a 2:1 ratio, and episodes of silence lasting more than 5 s occurred in only 35% of video-clips (usually only once).

**Table 1:** Mean frequency of utterances (SD) and ratio of affective to cognitive type (%)

	Cognitive	Affective	Ratio (%)
Overall	140 (55)*	13 (11)	10
Male	178 (55)*	13(10)	7
Female	133 (44)	19 (12)	15
Physician	163 (53)*	16 (11)	11
Nurse	90 (22)	14 (5)	17

*p < 0.01 vs affective, female or nurse. Frequency is per simulation

## Discussion

Healthcare cost containment and quality improvement are critical challenges, especially at the EOL. Overall rising economic burden is disproportionately high before death with decreasing efficacy of healthcare technologies. Palliative care, often sorely missing at the EOL, has been shown to improve quality at lower costs [50]. These issues are important for healthcare policy and raise questions about appropriate training of the workforce [1].

**Fig. 1:**
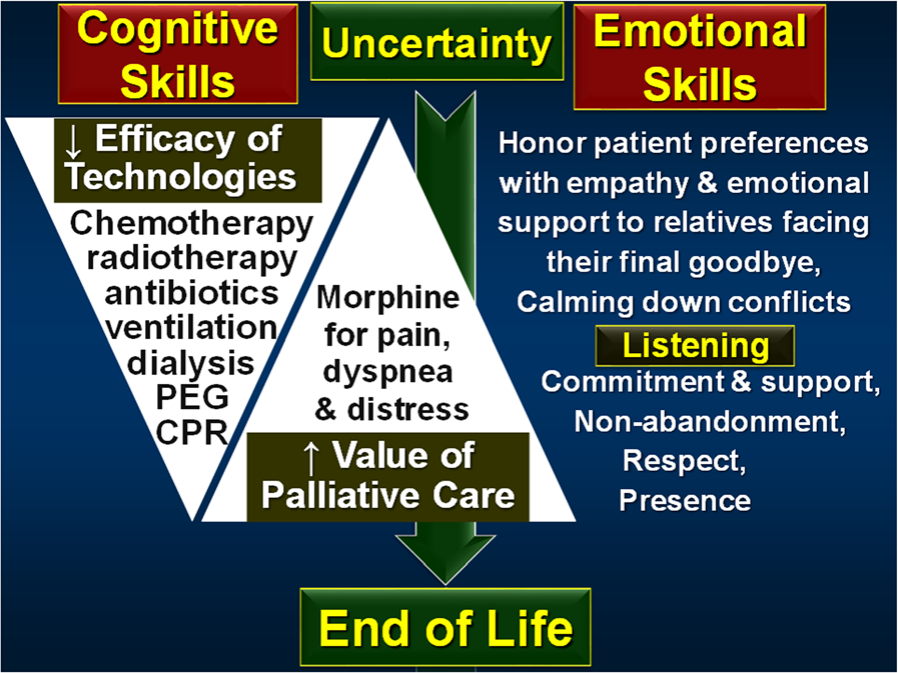
Model emerging from workshops discussions: Uncertainty often precedes EOL, with progressively reduced efficacy of technologies and increased value of palliative care. Concomitantly, emotional skills become increasingly more appropriate than cognitive skills to take care of needs of patients and families. Simulation-based training can improve those skills

The present study explores barriers in providing optimal care at the EOL using analysis of simulations and discussions at a training workshop for hospital staff. The themes emerging corroborate previous literature [1] including lack of training, knowledge, and time, overuse of technology, uncertainty about optimal decision-making, poor skills for communication and teamwork, and lack of experience at eliciting preferences for EOL care and at handling conflicts or dilemmas. RIAS-based analysis of video-clips showed dominance of cognitive over emotional utterances, and talk over listening. It seems as if to avoid confronting the affective burden of death, physicians keep busy with cognitive activities: physiological changes of dying, final diagnostic categories, biomedical options (tubing, antibiotic or other technologies) and legal issues – neglecting the affective needs of patients & families. An emerging model is shown in Fig. 1.

Beyond outlining challenges of the complex learning called forth by EOL care, group discussions and shared reflections about the simulation exercises raised potential solutions. Near death, uncertainty and dilemmas about decision using rational biomedical models, suggest alternative schemes based on emotional intelligence, listening and respect (as shown on the right-hand side of Fig. 1). A Chief of Medicine said at one workshop, “EOL often challenges me first whether I’m able to acknowledge I don’t know, and then, if I don’t know, whether I am able to listen.”

Indeed, family satisfaction at meetings about EOL care may be higher when physicians talk less and listen more [62, 63]. Dr. Susan Block says that “if physicians talk more than 50% of the time, they talk too much” [2, p.182]. The value of silence [64], listening [65], presence [66] and stillness as facilitator of deeper understanding [67] is not recognized by teams commonly enough. At conclusion of workshops, participants often agreed that at EOL “The gift of silent communion is the greatest gift you can give someone.” [65] Yet listening may be especially challenging for professionals with experience and authority [68]. One participant reported having heard from three patients at a leading academic medical center, “The only person in the team who still knows how to listen is the janitor”. Hierarchical structures collapse facing death. The silent presence of a warm, non-judgmental creature such as a cat can provide moving comfort at EOL [69].

An additional intrinsic value of listening may be for the clinician. Exquisite empathy can help prevent clinician’s burnout [70]. According to Rogers, listening is associated with personal and professional growth [71]. Young and others [72] describe mutuality of the healing process. Kubler-Ross wrote, “You may never admit it, but they [the dying] are *your* therapists, they are a gift to *you*” [73]. Major General Doron Almog said about his son who never spoke a single word (because of severe autism and mental disability): “He was the greatest teacher of my life” [74]. Several senior physicians (including an ICU Chief) mentioned at workshops that the most moving *thank you* letters they had ever received were not from people they saved but from families who had appreciated their humane presence at EOL. As Gawande concludes, “I never expected that among the most meaningful experiences I’d have as a doctor—and, really, as a human being—would come from helping others deal with what medicine cannot do as well as what it can” [2].

More than ever, in the currently alienated, overworked, and throughputs-oriented for financial survival, medicine may benefit from “time out” off technology: “It is an act of profound humility (…) to listen to the voices and silences of the children and adults for whom we care, and, in doing so, to approach a more-mindful medicine.” [64]. EOL may provide healthcare workers an opportunity to regain the value of human connection and, as suggested by philosopher E. Levinas, to retrieve their own meaning from the responsibility inferred from meeting the Other’s face [75]. Direct gaze between creatures stimulates oxytocin on both sides [76], suggesting reciprocity in the well-being induced by connection. Authenticity helps clinicians in caring at EOL [77–79] but may not be teachable by formal medical education [29] unless we understand it is key for our own satisfaction and spiritual growth.

Before her death at age 54, psychologist Marianne Amir wrote, “The aim of the health care team should be to create a secure environment of unconditional trust that patients can rely on to mediate between their inner world and the outside reality—an environment similar to that of maternal holding [referring to Winnicott’s work].” [80] Did she mean that, as at the beginning of life, EOL reminds us about the value of love?

### Limitations

Our study has significant limitations. First, the participants who came to the workshops may have had heightened interest in the issue of EOL, yet with significant challenges, which may be worse or different for other field clinicians who did not participate. Their self-evaluation is subjective and short-term. Second, our professional actors gave more authentic responses than “standardized patients” do with fixed repertoires, but the artificial setting barely reproduces the nuances of sensitive EOL communications with patients and families. Nevertheless, the workshop demonstrated room for improvement of skills to handle conversations, conflicts and dilemmas. Third, our study may not be generalizable to countries outside Israel – although the challenges observed remind those described in recent English literature [1].

A word of caution is warranted since our research vantage point used a participatory inquiry paradigm, which may be unfamiliar to many readers as it was new to us. According to value of the researcher’s reflexivity inherent to this paradigm, we attempted to describe as faithfully as possible the development of our own progress in understanding issues involved in EOL care, both in the results and in the discussion. Postmodern qualitative inquiry and action research are subjective, participatory, flexible, iterative, and context sensitive with explicit expression of dynamics of the researchers’ own views [81–84] including in the domain of healthcare [85]. Although our own conclusions may not apply elsewhere, we believe the process itself of deliberative dialogue between researchers and practitioners is reproducible and valuable, as it leads to interactive and collaborative learning [36].

Can simulation-based exercises improve handling EOL care? Studies show efficacy of simulation to enhance skills mostly among residents [13–18, 86, 87], but may fail to improve practice [19] without field implementation and on-going supervision [88] by senior workforce. It is challenging to have chiefs of service participate in a training workshop, let alone to change attitudes and behaviors ingrained by years of practice. Our experience with organic teams provided an occasion for senior physicians to observe residents and nurses in what they do often alone on shifts and weekends, and realize the needs for improvement. Conversely, simulations gave a chance for junior teams to see role models in action. In addition, the exchange between senior people from different institutions provided an opportunity for reflection, cross-fertilization and learning about options for better practice. Our initial intention targeted communication skills for EOL care (as previous work [11–18]), but as the project evolved, we became aware of deeper issues of competency and teamwork culture. Changing adult behavior for quality improvement may benefit from brainstorming, interactive learning and unlearning [30], listening for understanding [89] and mimicry [90]. More research needs to explore the value of simulation-based deliberative practice with providers in handling quality of care challenges and complex organizational learning [91]. This activity relates to what has been described in the literature as “evaluation capacity building” defined as a sustainable evaluation practice where members continuously ask questions that matter, collect, analyze, and interpret data, and use evaluation findings for decision-making and action [91]. This dynamic participatory paradigm may be more valuable than formally collected quantitative and qualitative static data most often used in reporting quality improvement projects. This mode of constructive cooperative exercise may be a framework for building organizational learning capacity with potential value in other challenges of quality in healthcare.

### Unexpected outcomes

A series of unanticipated consequences emerged from our project: One participant conducted a study of preparedness to EOL at nursing homes (eventually published [92]) that was presented at the Ministry of Health and called forth a national survey of preparedness to EOL at hospitals. As a result, many institutions enacted guidelines and set up palliative care units. Participants spread by word of mouth the value of training for EOL care – resulting in demands for workshops from different disciplines: intensive care, dialysis, oncology, emergency and family medicine. Electronic media (including TV channels), newspapers and magazines covered the topic of EOL care with reference to our workshops. We are invited each year to present insights from our project in lectures at dozens of national professional conferences, palliative care courses, research seminars, and institutional staff meetings as well as at general public audiences. While we cannot determine causality, coverage by media and public discourse led in recent years to the erection of several national committees for improved policy, training and regulation of EOL care.

## Conclusion

Simulation-based training of healthcare providers is an interesting and promising method to improve quality of EOL care. We observed an unexpected ripple effect manifested as national surveys, new palliative care services and expansion of EOL care education to other professional areas. Training a critical mass of staff providers may be valuable to induce a cultural shift in EOL care.

## Additional files

Additional file 1: Appendix A: Additional information on the procedures and the scenarios of the workshops. (DOCX 31 kb)

Additional file 2: Appendix B: Data from questionnaires filled out by participants at the start of workshops (Table S1), immediately at its end (Tables S2 & S3) and several months later (Table S4) (DOCX 26 kb)

Additional file 3: Appendix C: Specific lessons from the scenarios (DOCX 24 kb)

## Abbreviations

EOL: End of Life
MSR: Israel Centre for Medical Simulation
RIAS: Roter Interaction Analysis System
RN: Registered Nurse

## Footnotes

1 The steering committee was a self-appointed team of senior internists (MB, MF, AR) who initially supervised a resident in Medicine (YL) for her six-month research project (required for her certification in Internal Medicine) and included a first set of six workshops. The mandate was as mentors for this research project (as defined by the rules of the Israeli Medical Association for those research projects done as part of board certification) and the team members worked on a voluntary basis. After the completion of this project and the positive feedbacks we received from the participants, we decided to continue to operate as the steering committee for the full project described in this MS. For the workshops designed for ICU teams, Professor Charles Sprung convened an international group of intensive care experts (mostly from Europe and from the USA) to define goals, choose relevant scenarios and review survey questionnaires.

2 Deliberate practice is defined as repetitive structured activity to improve performance - key to expertise. Essential components include motivation of the learner, well-defined exercises, informative feedback and evaluation. Research shows that simulation-based education with deliberate practice yields improved results.

3 As of August 2017, we have conducted 127 workshops for 1710 healthcare providers.
