# Supplementary material for: What can we learn from simulation-based training to improve skills for end-of-life care? Insights from a national project in Israel
Source: Isr J Health Policy Res. 2017 Nov 6;6:48. doi: 10.1186/s13584-017-0169-9 (PMC5674237; doi:10.1186/s13584-017-0169-9)
Supplement: Supplementary file 1 — Appendix A: Additional information on the procedures and the scenarios of the workshops. (DOCX 31 kb) [file 13584_2017_169_MOESM1_ESM.docx]

**Appendix A as online supplemental material**

**Additional information on the procedures of the workshops**

The workshops, held at the Israel Center for Medical Simulation (near Tel Aviv), ran for seven hours, starting with an online survey on knowledge, attitudes and behavior regarding EOL care, while coffee and cookies were served and each participant received a name badge. In a round of introductions, everyone (including the group moderators) was invited to tell his or her name, worksite, function, seniority and, in order to break the ice, to say something personal, such as hobby or alternative profession if not in healthcare. Explanations were given on the process of the simulation exercises and the debriefing, and the participants were requested to sign an informed consent to the video recording.

A 45-min presentation discussed challenges of EOL care based on current literature, the principles of the Israeli Dying Patient Act including the obligation to alleviate suffering and to support families, and the skills expected to be practiced and improved at the workshop, such as listening, respect, empathy and emotional intelligence. After realizing the challenges for the participants through the initial runs of workshops, we added in the introductory lecture two tips: (1) How to elicit preferences for EOL care, (2) How to handle the time constraint (set at 7 min) of the simulation exercises. The first tip was to suggest nominating a proxy, “In case you would not be able to speak, who do you wish would speak on your behalf?” followed by “Have you spoken to him?” This would allow raising a delicate topic in a more sensitive manner than saying, “Your health condition is deteriorating and it’s time we discuss EOL preferences” – a tactic many providers felt very uneasy to use. The second tip presented research evidence[^1^](#_ENREF_1)^,^ [^2^](#_ENREF_2) showing that empathy is effectively expressed in less than one minute and is much faster than cognitive messaging.

After the introduction, the participants entered the simulations area either in the exercise room to face the actor, or in an adjacent corridor with one-way window and earplugs to watch the session. A written script on the door explained for the participant the situation he or she was about to meet (see below a short description of the scenarios). Three exercise rooms ran in parallel with different scenarios for two rounds in sequence allowing each participant to act or to watch. Two minutes before the end of the exercise, an announcement allowed participants to prepare for closure. After the exercise, both participants and actors filled out an evaluation form (to promote self-reflection) and the active participants re-enter the room for a feedback from the actors. After a 45 min shift, the participants gathered back in the main conference room for a 90 min debriefing session, based on audiovisual recordings of their performance. At debriefing, trainees receive constructive feedback from expert instructors and from peers. After a noontime break with refreshment, a second set of simulation exercises took place followed by debriefing – similar to the morning session but with different scenarios.

On average 14 (range 10-18) people attended each workshop. Each participant entered at least one or two simulation-based exercises and observed peers in several other scenarios. After a pilot run of workshops, we tried to have a physician and a nurse exercise together. It turned out that this type of combined training was useful for promoting teamwork, continuity of care and cross-fertilization between disciplines. We structured workshops to run most exercises with two trainees together (typically a physician with a nurse or a social worker) and sometimes three - giving more opportunities for participants to exercise.

At end of the workshop, we presented a summary of main lessons concluding with recommendations. We gave everyone a DVD with teaching materials (literature and presentations) and a personal copy of Kubler Ross’s book *Death is of Vital Importance* (translated in Hebrew), with the following quoted sentence as dedication: “You may never admit it, but they [the dying] are *your* therapists, they are a gift to *you*”.[^3^](#_ENREF_3) Last, we conducted a round of reflections where each participant was invited to share a personal take-home message – which quite often was “I need to listen more”. Before leaving, the participants filled out an online evaluation form.

**Short description of the scenarios at workshops for internal medicine and geriatrics**

**Scenario 1: Eliciting EOL care preferences from a patient.**An 85 year-old patient with chronic cardiac and lung diseases is now about to be discharged from hospitalization. He had recurrent hospitalizations during last year due to acute exacerbations and an actor plays this patient’s role. The workshop participant should elicit values and preferences for EOL care, discuss with him choices and think about decisions in case of respiratory failure, with the options for documenting his preferences and/or mandate a proxy – applying tools from the literature.[^4^](#_ENREF_4)

**Scenario 2: Eliciting EOL care preferences from a patient’s relative.**A 78 year-old patient with advanced dementia is in the hospital because of pneumonia. The participant meets an actor who plays his son’s role. The challenge is to elicit his father’s preferences for EOL care with empathy, listening to the son without asking him to make the decision (the strategy recommended in the Dying Patient Act[^5^](#_ENREF_5)).

In 2013, we added a question addressed by the actor to the trainer towards the end of this scenario, “What would you do if it were your father?” We included this question after reading a relevant publication on the significance associated with this daunting personal query: “Sharing a little piece of ourselves with patients and families humanizes us at a time when they need us to be human and sends the message that we are all in this together.”[^6^](#_ENREF_6)

**Scenario 3: Handling conflicts between siblings.**An 87 year-old semi-comatose woman with chronic multi-organ failure and respiratory distress presents a dilemma whether to intubate her. Her son, of secular background, is her direct caregiver and believes she would not have wanted artificial ventilation. Her daughter, of ultra-orthodox religious background, who lives with her eleven children in a remote site and rarely visits her mother, insists that everything should be done to save her. The trainee should handle the conflict between the actors who play the siblings – applying tools from the literature.[^7^](#_ENREF_7)

**Scenario 4: Handling conflict between the team and a relative who requests, “Do everything”.**An 89 year-old woman with from advanced dementia, heart failure and end-stage renal disease, is in the hospital because of sepsis. The team and the family, after discussion of options and review of previously elicited patient’s preferences, have reached the decision of comfort care. On a weekend, one son arrives from Australia, very agitated, having not seen his mother for years. He requests, “Do everything” and threatens for legal action, saying he is a lawyer expert in medical malpractice. In the simulation, participants meet an actor who plays this son. The challenge is to handle the conflict, avoiding being judgmental or overreacting to the threat, and to support with empathy.

**Scenario 5: Handling conflicts within the team.**A 75 year-old patient severely disabled from multiple cerebrovascular events, heart failure and diabetes is once again in the hospital with a large pneumonia. The family asked the team “without heroic interventions” to respect the patient’s values and preferences. The team decides for comfort measures only. A young physician who comes to be on duty in the ward disagrees with this decision. The participant meets an actor who plays the role of the resident who argues for full interventional treatment in this case, including intubation and CPR if necessary. The challenge is to handle the conflict, with respect to the young peer’s values, and to support him with empathy. The rationale for this scenario is the understanding of the importance of listening to diverse opinions in EOL ethical dilemmas, to strengthen teamwork and reduce burnout. [^8^](#_ENREF_8)

**Scenario 6: Explaining whether to put in a feeding tube.** An 86 year-old woman is in the hospital with dementia, poor eating, aspiration, weight loss & bedsores. She had previously expressed wish against tubing. Her son, proxy, is deeply troubled because on one hand he would like to respect his mother’s preferences. On the other hand, he is serious concerned by letting die from starvation the mother who had always fed an entire family with so much love. The workshop participants should lead an empathic conversation with a balanced explanation on benefits and harms of options, including explaining the lack of hunger, the limited efficacy of tube feeding and the value of comfort feeding. The basis for the challenge literature showing the need to support relatives in decision regarding feeding at EOL.[^9^](#_ENREF_9)^,^ [^10^](#_ENREF_10)

**Adapted scenarios in workshops for nursing homes, dialysis and intensive care units, and additional settings**

Over the years, the workshops have evolved to receive participants from different fields of practice. We adapted the scenarios for better fit of EOL care challenges in consultation with practitioners from those fields. We also added new scenarios as described below.

1. **Nursing homes**

One scenario described an elderly female indweller of a nursing facility with progressive dementia, bedsores and recurrent aspirations. The team and the family, after review of previously elicited patient’s preferences, has reached the decision of comfort care. One son, a lawyer, arrives from abroad, having not seen his mother for years. He requests, “Do everything” and threatens for legal action. In the simulation, participants meet an actor who plays this son and attempt to handle the conflict.

In another scenario, one of the senior nurses at a nursing home, has been providing dedicated care for several years to an elderly who has become progressively incapacitated because multiple severe health conditions. He is now poorly responsive, with a high fever, low blood pressure and oliguria. The nurse is very disturbed by the team decision not to transfer the patient to the ER. In the simulation-based exercise, the participants meet an actor who plays this nurse. The challenge is to handle the conflict and to support with empathy.

A different scenario describes a frail elderly resident of an assisted living facility who has become more and dependent. The team has reached the decision to transfer the resident to a different section of the facility with skilled nursing. A son opposes the decision and in the exercise, the participants meet the actor who plays his role.

In another scenario, a resident of a nursing home is complaining about the policy of the facility to have shared social activities with disabled people from the skilled nursing section. He says that yesterday he enjoyed the classic music concert but the sight of patient on a wheelchair with drooling around his nasogastric tube spoiled his pleasure. The exercise is to meet the actor who plays this role and handle the conflict.

In a final scenario describes the setting of a dying patient at a nursing home and his neighbor in an agitated delirium. The exercise challenges the participants to handle the situation with sensitive care and to practice support of EOL palliation for patients and the surroundings.

1. **Dialysis units**

One scenario describes a patient with dementia and multiple organ failure who has been on dialysis for several weeks – with no sign of improvement. His wife says to the nurses that he would have wanted to be in such a condition. In the simulation, two participants (dialysis nurses) meet an actor who plays the role of the physician in the unit, adamant to continue the dialysis.

Another scenario depicts an elderly diabetic woman on chronic dialysis and peripheral vascular disease, after leg amputation and now with severe ischemia in the other leg – for which she refuses amputation recommended by the orthopedic surgeon. The dialysis team, in the simulation-based exercise, meets an actor who plays the role of this patient.

A final scenario describes a patient on chronic peritoneal dialysis because of failure of vascular access for hemodialysis and after several rejected kidney transplantations. He suffers from severe pruritus, refractory to all therapies. He is desperate, irresponsive SSRI’s and wants to stops dialysis. The challenge for the team is to face a patient where all technologies have failed.

1. **Intensive care units**

In this setting, we added the challenge of discussing the option of organ donation in a case of brain death. The scenario was built in collaboration with the Israel Center for Organ Transplantation and we discussed at debriefing the proposed tools. [^11^](#_ENREF_11)

A scenario of conflict between healthcare providers described a surgeon who wants to operate for the sixth time a patient with complicated course after a Whipple’s procedure for pancreatic cancer, polymicrobial sepsis with resistant bugs, leaking anastomoses and circulatory shock. The participants face an actor who plays a rough and stubborn surgeon who insists on taking the dying patient to the OR – saying, “I promised the patient I will save him”.

1. **Family medicine**

For this setting, we included the challenge of having to deliver bad news to a middle age woman who did imaging studies for back pain that revealed widespread metastases.

Another scenario described an elderly widower coming to the clinic because of increasing pain from metastatic cancer. His son enters first and begs the physician not to mention his father the diagnosis of cancer (“my father does not know he has cancer”). If the participant asked the actor patient, “What do you think you suffer from?” The actor would answer, “I know I have the same disease my wife died from – cancer”.

1. **Emergency room**

In this setting, we added the challenge of having to deliver bad news to a father that his 15-year-old just died from a bike accident.

Another scenario was about a young woman returning to the ER because of intractable suffering from disseminated refractory cancer – after failure of multiple aggressive treatment protocols by oncologists. Her husband, in tears, says, “She has had enough” and requests euthanasia from the physician. Next to him, her mother sobs quietly. The challenge for the participant is to offer palliative sedation.

1. **Multidisciplinary general hospital setting**

We also developed an “[on wheels](https://eng.msr.org.il/msronwheels)” version of the workshop for a general hospital in the periphery of the country – traveling with the actors, the logistic team and video equipment. The purpose of this workshop was to train multidisciplinary teams avoiding them the need to travel to the simulation center in Tel Aviv.

In this version of the workshop, we adapted and combined scenarios from the above descriptions to fit practice of different wards (geriatric and internal medicine, surgical, gynecology, urology, ICU and ER).

**References**

1. Ambady N, LaPlante D, Nguyen T, Rosenthal R, Chaumeton N and Levinson W. Surgeons' tone of voice: A clue to malpractice history. *Surgery*. 2002; 132: 5-9.

2. Fogarty LA, Curbow BA, Wingard JR, McDonnell K and Somerfield MR. Can 40 seconds of compassion reduce patient anxiety? *Journal of Clinical Oncology*. 1999; 17: 371-9.

3. Kübler-Ross E. *Death is of vital importance: on life, death and life after death*. Station Hill Press, 1995.

4. Bernacki RE and Block SD. Communication about serious illness care goals: a review and synthesis of best practices. *JAMA Internal Medicine*. 2014; 174: 1994-2003.

5. Steinberg A and Sprung CL. The dying patient: New Israeli legislation. *Intensive Care Medicine*. 2006; 32: 1234-7.

6. Korones D. What would you do if it were your kid? *New England Journal of Medicine*. 2013; 369: 1291.

7. Back AL and Arnold RM. Dealing with conflict in caring for the seriously ill: “it was just out of the question”. *JAMA*. 2005; 293: 1374-81.

8. Kearney MK, Weininger RB, Vachon ML, Harrison RL and Mount BM. Self-care of physicians caring for patients at the end of life: “Being connected... a key to my survival”. *JAMA*. 2009; 301: 1155-64.

9. Golan I, Ligumsky M and Brezis M. Percutaneous endoscopic gastrostomy in hospitalized incompetent geriatric patients: poorly informed, constrained and paradoxical decisions. *The Israel Medical Association Journal*. 2007; 9: 839.

10. Teno JM, Mitchell SL, Kuo SK, et al. Decision‐making and outcomes of feeding tube insertion: A five‐state study. *Journal of the American Geriatrics Society*. 2011; 59: 881-6.

11. Ashkenazi T and Klein M. A practical Israeli strategy for appealing for organ donation. *Progress in Transplantation*. 2013; 23: 173-9.
