# Supplementary material for: What can we learn from simulation-based training to improve skills for end-of-life care? Insights from a national project in Israel
Source: Isr J Health Policy Res. 2017 Nov 6;6:48. doi: 10.1186/s13584-017-0169-9 (PMC5674237; doi:10.1186/s13584-017-0169-9)
Supplement: Supplementary file 2 — Appendix B: Data from questionnaires filled out by participants at the start of workshops (Table S1), immediately at its end (Tables S2 & S3) and several months later (Table S4) (DOCX 26 kb) [file 13584_2017_169_MOESM2_ESM.docx]

**Appendix B as online supplemental material**

**Data from questionnaires filled out by participants at the start of workshops (Table 1), immediately at its end (Tables 2 & 3) and several months later (Table 4).**

Closed questions used a Likert scale. Unless indicated otherwise, participants answered the questionnaires online at computer stations of the simulation center. The survey software (Nemala Proactive Dialog Solutions, TOP GROUP, Tel Aviv) collected, analyzed and yielded output data in an excel format. The questionnaires were developed by steering committee members. In pre-testing surveys, at-face validity was examined by verbal inquiring a sample of responders, and, when needed, the wording was improved until the understanding of question was satisfactory. In a pilot survey, reliability was measured for the same questionnaire given twice, two weeks apart, to the same responders: Cronbach alpha ranged between 0.6-0.8.

**Table 1A Self-evaluation of skills for EOL care (N=752)**

| Question | % who answered: | | |
| --- | --- | --- | --- |
|  | “Not at all or a little” | “To a fair extent” | “To a large extent” |
| Do you have tools to discuss EOL care preferences with patients? | 44% | 43% | 11% |
| Do you have tools to discuss EOL care preferences with families? | 28% | 53% | 17% |
| Do you have tools to provide emotional support for patient and family at EOL? | 24% | 51% | 21% |
| Do you have tools to deal with family request to “do everything”? | 34% | 49% | 13% |
| Do you have tools to deal with conflicts within the family at EOL? | 61% | 30% | 5% |
| Do you have tools to deal with conflicts within medical staff members at EOL? | 31% | 51% | 14% |

| Question | % who answered: | | | |
| --- | --- | --- | --- | --- |
|  | “Never” | “Rarely” | “Often” | “Nearly always” |
| How often do you use, in patients at EOL, invasive, life-sustaining procedures that you feel are useless? | 8% | 34% | 47% | 8% |
| How often do you perform, in your ward, “slow codes” or “show codes”? | 19% | 56% | 19% | 2% |
| How often do you provide to others treatment you would not want for yourself in the same situation? | 6% | 21% | 58% | 9% |
| How often do you use optimal palliative care with significant alleviation of suffering in patients at EOL (including use of morphine)? | 1% | 23% | 57% | 15% |
| How often do you use morphine to alleviate dyspnea? | 4% | 35% | 51% | 6% |
| How often do you approach the family of a patient at EOL for the main purpose of listening (as opposed to giving them information)? | 2% | 26% | 46% | 22% |

**Table 1B Self-report of provider’s behavior relevant to EOL care (N=752)**

| Question | % who answered: | | | |
| --- | --- | --- | --- | --- |
|  | “Very low” | “Low” | “High” | “Very high” |
| Grade the quality of your conversations with patients and families regarding preferences for EOL care | 8% | 34% | 50% | 3% |
| Grade the degree of your satisfaction from the way decisions are being made at the EOL in your ward | 6% | 38% | 47% | 3% |

Note: Response rate for data in Table 1 was 70% because late arrivers to the workshop skipped the survey. In addition, we had to exclude 250 questionnaires for various reasons (different version of questions, missing answers, technical issues in retrieving data).

**Table 2 Rating of workshop by participants at the end of the day (N=976)**

| Question | % who answered | | |
| --- | --- | --- | --- |
|  | “Not at all or a little” | “Much” | “Very much” |
| Did the opening presentation help you? | 1% | 23% | 72% |
| Did the scenarios reflect reality? | 1% | 20% | 78% |
| Did the simulations help your communication skills for decisions at the end-of-life (EOL)? | 3% | 26% | 68% |
| Did the debriefing discussions help your communication skills for decisions at EOL? | 2% | 26% | 69% |
| Following the workshop, will you feel more comfortable initiating EOL discussions with patients and families? | 4% | 33% | 61% |
| Following the workshop, will your handling of these discussions going to be different? | 9% | 35% | 53% |
| Following the workshop, will your handling of EOL patients going to be different? | 10% | 33% | 52% |

Note: Response rate was 90% because some participants, in a rush at the end of the day, skipped the survey. In addition, we had to exclude 240 questionnaires for various reasons (different version of the questions, missing answers, technical issues in retrieving data).

| Question | % indicating item | |
| --- | --- | --- |
| In which area do you fill lack of knowledge or skills, for dealing with EOL? | “Dying Patient Act” law  Communication skills  Prognosis estimation | 44%  31%  17% |
| What are the barriers, in your opinion, to good quality care at EOL? | Lack of law knowledge or fear from law  Lack of palliative care skills  Fear from death or from dealing with death  Lack of time | 37%  28%  37%  20% |
| Recurrent comments at nearly each workshop | “Every physician and nurse should come to this workshop in order to improve EOL care.”  “Adaptation of workshop to other specialties is needed.”  “Heterogeneity of participants’ background (profession, seniority) was important for cross learning.”  “We need more of this kind of training!” | |

**Table 3 Perceived challenged and open comments by participants at the end of workshop (N=976)**

Note: Response rate as for Table 2

**Table 4 Late evaluation of impact from training - several months after workshop (N=210)**

| Topic of the question | % reporting | | |
| --- | --- | --- | --- |
|  | Improvement | No change | Worsening |
| Your willingness and capability to engage patients and families in discussion about preferences for EOL care | 80% | 19% | 0.5% |
| Initiation of a meeting with the family of a patient in critical condition | 66% | 34% | 0% |
| Combined meeting of nurse and physician with the relatives of EOL patient | 56% | 43% | 0.5% |
| Your degree of initiative to promote staff discussions regarding patients at EOL | 76% | 24% | 0% |
| Frequency of discussions at staff meetings on care dilemmas for EOL patients | 57% | 43% | 0.5% |
| Quality of discussions at staff meetings and with relatives of EOL patients | 62% | 38% | 0.5% |
| Decreased use of medical procedures with perceived low utility | 66% | 33% | 0.5% |
| Alleviation of suffering in EOL patients | 80% | 20% | 0% |
| On tube feeding, discussion of harms, unsure benefit and option of comfort feeding | 62% | 37% | 0.5% |
| Use of morphine to alleviate dyspnea at EOL | 68% | 31% | 1% |
| Your capability of handling conflicts surrounding EOL dilemmas | 78% | 22% | 0.5% |
| Approaching patients and families to listen, to respect and to be with them | 77% | 23% | 0% |
| Elicitation and actual documentation of patients’ preferences for EOL care | 70% | 29% | 0.5% |
| Promoting written advanced directives and proxy appointment among patients (for instance at discharge) | 59% | 40% | 0.5% |
| Your level of apprehension from coping with patients and families in the last hours before death | 76% | 24% | 0% |
| Your perceived capability to support other team members who have difficulties handling EOL situations | 80% | 20% | 0% |
| Your perceived professional satisfaction from supporting and escorting with respect separation and loss at EOL | 76% | 24% | 0% |
| Your level of knowledge regarding the “Dying Patient Act” | 87% | 13% | 0% |
| In invited open comments, a majority of respondents indicated the following significant changes: “better care of EOL patients as a team”; “understand and reflect more deeply about EOL”; “more use of morphine and of palliative care”; “approach families, initiate discussions and support”; “help a patient die in dignity”; “respect patient’s wishes”; “elicit preferences for EOL care”; “listen more and respect more”; “better alleviation of suffering and avoidance of invasive treatments”; “better communication”. | | | |

Note: We restricted this survey to internal medicine. Response rate was 30% as we encountered difficulty in reaching participants several months after the workshop. Limitations include self-evaluation from a small and potentially biased sample of responders. We also conducted two focus groups with 50 physicians and nurses, who had participated in a workshop 3-6 months earlier and the themes that emerged from the discussion, were similar to those indicated in open comments at the bottom of the table. For instance, one nurse reported using more morphine despite reluctance of others in her team. A geriatrician reported a systematic attempt in his ward to elicit patient’s preferences for EOL care. One resident reported more discussions of EOL ethical dilemmas at staff meeting facing a conservative chief of medicine by a critical mass of physicians who attended the workshop. Two physicians recounted their support to allow discharging hospital patients to die peacefully at home and the remarkable gratitude their received from the families after the death. These narratives together with the data in the table suggest a shift in the approach to EOL for at least some participants of the workshops.
