# Supplementary material for: What can we learn from simulation-based training to improve skills for end-of-life care? Insights from a national project in Israel
Source: Isr J Health Policy Res. 2017 Nov 6;6:48. doi: 10.1186/s13584-017-0169-9 (PMC5674237; doi:10.1186/s13584-017-0169-9)
Supplement: Supplementary file 3 — Appendix C: Specific lessons from the scenarios (DOCX 24 kb) [file 13584_2017_169_MOESM3_ESM.docx]

**Appendix C as online supplemental material**

**Specific lessons from the scenarios**

*Two scenarios addressed eliciting preferences for EOL care from a patient or from a relative.*

The most challenging scenario turned out to be eliciting preferences for EOL care from an elderly actor with chronic illnesses and frequent hospitalizations. Participants admitted, “Having never learned how and having little experience in conducting such conversations.” They felt highly uncomfortable, even facing an actor, explaining that limited prognosis warrants advance care planning. Some felt inappropriate to raise such an issue at recovery from an acute exacerbation (setting of scenario) as it might “negate hope”, and they would refer the challenge to the GP (who show interest[^1^](#_ENREF_1)). Successful approaches included open questions unrelated to prognosis, such as “what are your concerns?” or “in case you would not be able to talk, who you would suggest to speak on behalf of you?” If the participant proposed advanced directives, the actor asked, “Will you respect my wishes? In my recent hospitalizations, you took blood tests, sent me to X-rays, gave me medications - did anyone ask for my opinion?” The actor (in real life also a patient) suggested that respecting autonomy should not wait for EOL. If a participant explained options such as intubation and CPR, the actor replied, “How do I know? I’m a farmer; I know to grow tomatoes”. At debriefing, we discussed the challenge of shared-decision making, applying tools such as video clips.[^2-4^](#_ENREF_2) Participants brought up the issue of instability of preferences and difficult affective forecasting;[^5^](#_ENREF_5) we suggested aiming at eliciting values (rather than predilections for tubing[^6^](#_ENREF_6)), committing ourselves to respect those values and sending a message of non-abandonment.

Participants felt more at ease in a different scenario that required eliciting preferences for EOL care from the relative of an unconscious patient. Yet, they sometimes would confuse the nuance between asking families “what do you want us to do?” and a more appropriately worded question: “if he were here with us, what he would have said and wished for?” In response to actor’s surprise at bad news (an emergency department physician had re-assured him “he will get better with antibiotics”), most participants would bio-medically explain the deterioration, although *cognitive* argumentation may not be best to address denial - an *affective* state of mind. At discussion, we proposed tools aimed at enhancing trust: listening, respect, humility and apologies for errors (see Box 1 in reference[^7^](#_ENREF_7)). At the end, the actor would ask participants, “Please forgive me for asking, but if it were your dad, what would you do?” At debriefing, participants said they often hear similar questions at work and while some felt uneasy with “intrusion into their private life”, others responded with candor, for instance that “they would respect preferences and alleviate suffering”. At discussion, participants accepted that such a question is a call for humanness, to quote from reference[^8^](#_ENREF_8): “Sharing a little piece of ourselves with patients and families humanizes us at a time when they need us to be human and sends the message that we are all in this together”.

*Three scenarios addressed conflicts in decision making for EOL care, within the family, within the team, or between a relative and the team.*

One scenario presented a vehement conflict between siblings regarding care for their dying mother: the religious daughter requested to “do everything”, and the secular son, the patient’s proxy, asked for comfort measures only. Participants tended to take a side in the fight or appeared at lost. Some asked, “What do you know about your mother’s condition?” and “What do you mean by doing everything?” A few participants showed empathic listening to both sides and calmly explained, “I can see how hard this is for both of you, and how deep is your love for Mom. I guess she would wish at this time you to respect each other and be in peace”. In one such instance, an actor cried and explained later, “I thought about my mother with advanced dementia, wishing I had met such an empathic physician in real life”. At debriefing we discussed tools to handle conflicts,[^9^](#_ENREF_9) acknowledging that while not taught in medical school, such skill is needed to support families, as requested by the Israeli Dying Patient Act.[^10^](#_ENREF_10)

A different conflict scenario presented a young resident (actor) who disagrees with the staff’s decision for DNR and meets a senior physician (participant). The challenge was handling a conflict within the team and respecting dissident opinions. Some participants strived at convincing the resident with rational arguments (“respect to team decision and to family’s expectations”) while ignoring the ego element in the conflict. Others listened with patience and offered support, “You can call me anytime and I will come” and a few thanked the resident for his independent viewpoint. One Chief said, “Can you tell me, perhaps you had an experience that accounts for your position?” to which the actor improvised a story about “his deceased mother agonizing with a dreadful cancer while he was taking issue with his father who said she had suffered enough and to let her go.” The discussion focused on the value of airing dissident views as a team culture for both safety[^11^](#_ENREF_11) and burnout prevention.[^12^](#_ENREF_12)

In yet another conflict scenario, the actor plays a lawyer expert in medical negligence, living abroad, who returns to visit his dying mother he has not seen for years. He rejects staff’s decision for comfort measures taken with the family, based on patient’s values. He demands to “do everything” and threatens to sue the physician. While participants said, “they are used to legal threats”, they felt uneasy and had difficulty handling the situation. Some attempted empathy to address the son’s guilt feelings; one physician said, “I apologize if I have hurt you” – to which the actor melted. At debriefing, a suggested approach to handle the conflict was to be non-judgmental and to address the affective storm with active listening (as discussed in Box 2 of reference[^7^](#_ENREF_7)). Giving support to an angry son (as requested by law[^10^](#_ENREF_10)) demands realizing the emotional burden and perhaps saying, “It’s good you were able to come; not everyone gets to arrive in time to say goodbye”.

*A final scenario addressed a dilemma about applying life support technology at EOL, exemplified by the controversial value of tube feeding in advanced dementia.*

The topic here was to discuss options for tube feeding with the son of an elderly woman with advanced dementia, recurrent aspirations, bedsores and poor eating - while a nurse had successfully given her to swallow a few spoons of cereal that morning. The son, who is the patient’s proxy, reports that his mother would not have wanted tubes. Participants offered a feeding tube “for nutrition, healing sores and preventing aspirations” without mentioning the need for restraints should the patient pull out the tube – as the actor was trained to ask. Others suggested continuing assisted oral feeding as long as possible, “As evidence does not support the efficacy of tube feeding.” At debriefing, lively discussions took place between protagonists of tube feeding (a Chief of Medicine said, “This is what we teach”) and other participants. A Chief of Geriatrics explained, “At life’s beginning, babies need frequent feeding for growth; ageing & dementia are associated with reduced caloric intake and no starving.” A notion emerging from discussions was that divergence of physicians’ opinions reflect differing interpretation of limited evidence (lacking randomized trials [^13^](#_ENREF_13)) and contribute to confusion perceived by families.[^14^](#_ENREF_14)^,^ [^15^](#_ENREF_15) Physicians need to admit lack of strong evidence regarding efficacy of tube feeding as for many technologies at EOL. The *process* of decision-making, respecting and supporting stakeholders, is more significant than the decision itself (based on physiological rationale or convenience to staff[^16^](#_ENREF_16)). A senior physician of a religious center (where tube feeding is the rule in advanced dementia[^17^](#_ENREF_17)), reported several months after the workshop, that now “he tries first understanding patient’s values & family’s wishes before explaining arguments supporting their choice.” Listening is key to improve decision-making in many dilemmas on life-support at EOL.

*Communicating bad news* was a simulation exercise included in specific workshops for teams from Family medicine and Emergency room as indicated in Appendix A. The challenge was felt to be difficult by both nurses and physicians, including younger physicians who quite often had received training as students with the SPIKES tool - an observation that resonates with the literature.[^18^](#_ENREF_18) We found useful in the discussion to watch the didactic [short video prepared by Diane Meier](The%20Human%20Connection%20of%20Palliative%20Care:%20Ten%20Steps%20for%20What%20To%20Say%20and%20Do).

1. Lifshitz G, Cohen MJ, Shmilovitz H, Brezis M, Lahad A and Ben-Yehuda A. Physician-Facilitated Designation of Proxy Decision-Makers: Family Physician Perceptions. *Israel Journal of Health Policy Research*. 2016; 5: 1.

2. Austin CA, Mohottige D, Sudore RL, Smith AK and Hanson LC. Tools to Promote Shared Decision Making in Serious Illness: A Systematic Review. *JAMA Internal Medicine*. 2015.

3. Butler M, Ratner E, McCreedy E, Shippee N and Kane RL. Decision Aids for Advance Care Planning: An Overview of the State of the Science. *Annals of Internal Medicine*. 2014.

4. Volandes A. *The Conversation: A Revolutionary Plan for End-Of-Life Care*. First U.S. edition. ed.: Bloomsbury, 2015. See https://[www.acpdecisions.org/](http://www.acpdecisions.org/).

5. Halpern J and Arnold RM. Affective Forecasting: An Unrecognized Challenge in Making Serious Health Decisions. *Journal of General Internal Medicine*. 2008; 23: 1708-12.

6. Schenker Y, White DB and Arnold RM. What Should Be The Goal of Advance Care Planning? *JAMA Internal Medicine*. 2014; 174: 1093-4.

7. Tulsky JA. Beyond advance directives: importance of communication skills at the end of life. *JAMA*. 2005; 294: 359-65.

8. Korones D. What would you do if it were your kid? *New England Journal of Medicine*. 2013; 369: 1291.

9. Back AL and Arnold RM. Dealing with conflict in caring for the seriously ill: “it was just out of the question”. *JAMA*. 2005; 293: 1374-81.

10. Steinberg A and Sprung CL. The dying patient: New Israeli legislation. *Intensive Care Medicine*. 2006; 32: 1234-7.

11. Wilson KA, Burke CS, Priest HA and Salas E. Promoting health care safety through training high reliability teams. *Quality and Safety in Health Care*. 2005; 14: 303-9.

12. Kearney MK, Weininger RB, Vachon ML, Harrison RL and Mount BM. Self-care of physicians caring for patients at the end of life:“Being connected... a key to my survival”. *Jama*. 2009; 301: 1155-64.

13. Sampson EL, Candy B and Jones L. Enteral tube feeding for older people with advanced dementia. *The Cochrane Library*. 2009.

14. Golan I, Ligumsky M and Brezis M. Percutaneous Endoscopic Gastrostomy in Hospitalized Incompetent Geriatric Patients: Poorly Informed, Constrained and Paradoxical Decisions. *IMAJ*. 2007; 9: 839-42.

15. Teno JM, Mitchell SL, Kuo SK, et al. Decision‐making and outcomes of feeding tube insertion: A five‐state study. *Journal of the American Geriatrics Society*. 2011; 59: 881-6.

16. Shaulov A, Frankel M, Rubinow A, Maaravi Y and Brezis M. Preparedness for end of life – a survey of Jerusalem district nursing homes. *Journal of the American Geriatrics Society*. 2015; 63: 2114-9.

17. Clarfield AM, Monette J, Bergman H, et al. Enteral Feeding in End-Stage Dementia: A Comparison of Religious, Ethnic, and National Differences in Canada and Israel. *The Journals of Gerontology Series A: Biological Sciences and Medical Sciences*. 2006; 61: 621-7.

18. Brown R, Dunn S, Byrnes K, Morris R, Heinrich P and Shaw J. Doctors' stress responses and poor communication performance in simulated bad-news consultations. *Academic Medicine*. 2009; 84: 1595-602.
